# The Relationship Between Adult Attachment Orientation and Mindfulness: a Systematic Review and Meta-analysis

**DOI:** 10.1007/s12671-017-0733-y

**Published:** 2017-05-24

**Authors:** Jodie C. Stevenson, Lisa-Marie Emerson, Abigail Millings

**Affiliations:** Department of Psychology, The University of Sheffield, Cathedral Court, Sheffield, S1 2LT UK

**Keywords:** Attachment, Adult attachment, Attachment anxiety, Attachment avoidance, Meta-analysis, Mindfulness, Systematic review

## Abstract

Mindfulness can be measured as an individual trait, which varies between individuals. In recent years, research has investigated the overlap between trait mindfulness and attachment. The aim of the present review and meta-analysis was to investigate the current evidence linking adult attachment dimensions to trait mindfulness dimensions, and to quantitatively synthesize these findings using meta-analyses. A systematic literature search was conducted using five scientific databases of which, upon review, 33 articles met inclusion criteria. Inclusion criteria were peer-reviewed journals and dissertations published in English that relied on quantitative methods using reliable and validated self-report measures where study participants were aged 16 years and older. Random-effects model meta-analytic procedures were used to investigate the relationship between both constructs. Cross-sectional studies found significant negative correlations between adult attachment insecurity, on either dimension (anxiety or avoidance) and both total mindfulness score and all five sub-dimensions of mindfulness (act with awareness, observe, describe, non-reacting, and non-judging), with the exception of a non-significant positive correlation between attachment anxiety and observe. The effect size of the relationships ranged from small to medium. The overall mean effect sizes were moderate (anxiety, *r*_+_ = .34; avoidance, *r*_+_ = −.28), with both attachment dimensions associated with lower levels of total mindfulness. Results are discussed in relation to theory and research. Implications for future research include the need to utilize longitudinal design to address causality and mechanisms of the relationship between these constructs.

Mindfulness is defined as the self-regulation of attention and the non-evaluative acceptance of one’s immediate experiences (Kabat-Zinn 1994); it can also provide a greater sense of compassion and kindness for oneself and others (Neff 2012). Mindfulness is a distinctive state of consciousness compared to that of typical cognitive processing as the individual allows sensory input, noticing it rather than comparing, evaluating, or ruminating about it (Brown et al. 2007). The term mindfulness has been used to describe (i) a psychological trait (dispositional or trait mindfulness), which varies between individuals (Brown and Ryan 2003); (ii) a particular state of awareness (Germer et al. 2005); and (iii) a contemplative practice (such as mindfulness meditation and mindfulness stress reduction techniques). Contemplative mindfulness practices allow the individual to access a particular state of awareness at the time of practicing. Dispositional mindfulness reflects an individual’s natural inclination towards a mindful way of being, and will likely influence their ability to engage in mindfulness practices and achieve a mindful state. In the development of questionnaire measures of dispositional mindfulness, Baer and colleagues (Baer et al. 2004, 2006, 2008) reported five facets: observing (noticing internal and external stimuli), describing (labeling one’s experiences), acting with awareness (attending fully to one’s activity, without “autopilot”), non-judging (refraining from evaluating one’s experiences), and non-reacting (experiencing one’s thoughts and feelings without needing to immediately respond).

A large body of research has demonstrated that as a contemplative practice, mindfulness has benefits on mental, emotional, and physical health, and can lead to increases in dispositional mindfulness (Greeson 2009; Keng et al. 2011; Brown and Ryan 2003; Baer 2015). In addition, dispositional mindfulness is associated with positive psychological outcomes including stress reduction, lower emotional reactivity, as well as increased well-being (Farb et al. 2010; Ortner et al. 2007; Carmody and Baer 2008). These positive effects are likely because mindfulness enables individuals to disengage from their automatic thoughts and behavior patterns and, in turn, fosters informed and conscious regulation as a means to promote positive functioning (Ryan and Deci 2000). Given the rapidly growing body of research on mindfulness, it is important that we are able to delineate the correlates and antecedents of mindfulness, for example, which individual difference factors might be related to, and indeed predictive of, dispositional mindfulness. In considering the antecedents of mindfulness, researchers have posited that, along with aspects of adaptive functioning, it is helpful to place mindfulness within a social context. Furthermore, researchers have proposed that the attachment theory provides the most appropriate conceptual framework with which to do so (Shaver et al. 2007).

Attachment theory (Bowlby 1969) postulates that adult attachment style, a trait-like pattern of affect regulation strategies, develops as a reflection of the sum total of experiences of being cared for in close relationships. As a result of these caregiving experiences, individuals develop an internal working model (Bowlby 1969, 1973) of the self, others, and relationships, that guide the manner in which we experience and deal with stress or threat (Bowlby 1982; Waters et al. 2002). Internal working models are essentially pathways of cognitive structures that reflect the cumulative perceptions of personal experiences with past attachment figures (Collins et al. 2004). Research has highlighted the influence that these working models have on information processing and interpersonal functioning, which include attitudes, emotions, affect regulation, and behavioral strategies (see Mikulincer and Shaver 2007; Shaver and Mikulincer 2002 for a review). More broadly, they influence the way we attend to and perceive information, from both internal and external sources. Two research traditions exist in adult attachment. The developmental tradition tends to focus on the role of maternal relationships in early life, and how these affect intergenerational (parenting) relationships with children, and mental health (Schore 2001). This research tradition often employs interview and narrative techniques to assess state of mind with respect to attachment (Shaver and Mikulincer 2002). The social, cognitive, and personality tradition tends to focus more on adult pair bond relationships, and hierarchies of attachment styles with a variety of current attachment figures (Collins and Read 1994). This research tradition often employs self-report measures of attachment style, in relation to romantic partners or close others generally, and the impact they have on a wide variety of outcomes associated with well-being and functioning (see Mikulincer and Shaver 2007, for a review).

Current conceptualizations of attachment style focus on two dimensions of attachment insecurity: anxiety about abandonment and avoidance of intimacy (Brennan et al. 1998). When individuals have repeated experiences of caregivers being sensitive and responsive to their needs, they score low in both anxiety and avoidance, reflecting a secure attachment style characterized by a balanced approach to support seeking and emotion regulation. Attachment needs are not denied or suppressed, and nor are they overwhelming. Those who experience caregivers who are inconsistently available and responsive score highly in attachment anxiety. Such individuals tend to engage in hyperactivation of the attachment system, characterized by increased efforts to seek proximity and protection, a hypersensitivity to signs of rejection, and excessive rumination on one’s own shortcomings and immediate relationship threats (Mikulincer and Florian 1998). Those who experience caregivers that are consistently rejecting or non-responsive score high in attachment avoidance, and tend to engage in deactivation of the attachment system, characterized by avoidance of proximity seeking, denial of attachment needs, and the suppression of signs of vulnerability (Mikulincer and Shaver 2003).

There are several points of contact between mindfulness and attachment security which were first identified by Ryan et al. (2007), and subsequently expanded upon by Shaver et al. (2007). Firstly, both constructs share similar positive effects on a range of outcomes related to well-being. Secure adult attachment and mindfulness have been linked to the same positive outcomes regarding one’s mental and physical health, along with more successful relationships, adaptive coping when faced with threatening stimuli, higher self-esteem, and increased self-regulation (Ryan et al. 2007; Shaver et al. 2007). Moreover, neurological studies have reported similar neural pathways for secure attachment, emotional self-regulation, and mindfulness (Gillath et al. 2005; Shaver et al. 2007; Siegel 2007). While much remains to be explored in regards to the neural correlates of attachment and mindfulness, the existing literature suggests that those neural structures governing executive functioning, emotional regulation, and attention are indeed associated with the constructs of attachment and mindfulness (Siegel 2007).

Secondly, there are parallels that can be drawn between secure attachment relationship experiences and Buddhist forms of mindfulness meditation in terms of accessing mental representations of security and bolstering mindfulness, and conversely, between insecure representations and hampered mindfulness efforts. Buddhist practice can involve accessing representations of acceptance by a loving Buddha, their teachings, and a community of fellow Buddhists, which is conceptually similar to attachment theory’s notions of secure base and safe haven provided by security-enhancing attachment figures (Shaver et al. 2007). Furthermore, when we consider attachment insecurity, it is easy to see how incompatible both avoidance and anxiety are with effective mindfulness practice. While mindfulness represents an open and accepting outlook with direct observations and a removed or decentered approach to thoughts and feelings, attachment anxiety leads to feelings of unworthiness, hypervigilance along with a hypersensitivity to rejection, and also increased levels of rumination (Mikulincer and Florian 1998). Conversely, attachment avoidance is characterized by emotion and thought suppression, a discomfort with close relationships, and avoidance regarding thoughts and feelings, more often towards those negative in nature (Mikulincer and Shaver 2003). Shaver et al. (2007) likened attachment anxiety to the “grasping” at or obsessing over, and attachment avoidance to the repression of, unwanted thoughts in meditative practice (Chödrön 2003). Attachment security, however, should enable an individual to approach their thought processes in a more balanced and forgiving way.

Thirdly, there may be a bidirectional relationship such that security-enhancing relationship experiences are likely to increase a person’s capacity for mindfulness and conversely, mindfulness meditation is likely to increase a person’s capacity for secure relationships. In light of the similarities between attachment security and mindfulness, and incompatibility between attachment insecurity and mindfulness, researchers have theorized about the relationship between these two variables. It is possible that an individual’s secure attachment may cultivate compassion for the suffering of others. Such compassion is also regarded as a product of mindfulness (Mikulincer et al. 2005; Brach 2003; Neff 2003). Ryan et al. (2007) emphasized three potential connections between secure attachment and mindfulness and the bidirectional nature of this relationship: (i) it is possible that they develop simultaneously in response to a caring, responsive, and comforting caregiver experience throughout childhood; (ii) they may both be related to attentive and securely attached relationship styles; and (iii) secure attachment and mindfulness may both be related to the development of adequate qualities and mechanisms to deal with stress.

Some researchers have attempted to assess directionality in this relationship. Ma (2008) reported that mindfulness partially mediated the association between increased attachment security and overall adaptive functioning, and that changes in mindfulness during therapy partially mediated the link between changes in attachment security and changes in adaptive functioning (Ma 2008). However, analyses presenting attachment security as the mediator between mindfulness and adaptive functioning were not presented, so it is not possible to be confident that attachment security is a precursor for mindfulness in these data, rather than the other way around. Further research speaks to the issue of directionality in the relationship between attachment and mindfulness. Rowe et al. (2016) primed mindfulness naïve participants with attachment security, self-compassion, or a neutral control, prior to them undertaking a taster session in mindfulness. Participants who received either the security prime, or the self-compassion prime, were more willing to engage in further mindfulness training. It is possible that these primes, including attachment security, made it easier for participants to successfully achieve a mindful state. But whether the practice of mindfulness could also enable participants to more successfully visualize attachment security is yet to be examined. Researchers have also investigated whether training in mindfulness could offset or ameliorate the negative impacts of attachment insecurity on relationships with others, such as children (Snyder et al. 2012). Further research is needed to examine this potential effect the other way around, i.e., whether activating a sense of attachment security could ameliorate the impact of having low trait mindfulness.

The potential for bi-directionality in the relationship between attachment security and mindfulness comes from the core qualities of mindfulness fostering a secure attachment, as well as a secure attachment fostering the development of mindfulness. Those individuals who exhibit a secure attachment style are likely to develop self-trust and also be trusting of others, to be easily placated and comforted when stressed, have a repertoire of effective coping strategies, and to be compassionate (Ryan et al. 2007). It is these qualities that are thought to allow individuals to pay attention, to be present to both positive and negative experiences, and to do so non-judgmentally, which are all key components of mindfulness.

While the directionality and mechanisms of the relationship between attachment and mindfulness are not yet well defined, there seems little doubt that these two constructs are very likely to be linked. Indeed, in the 9 years since researchers began to theoretically examine the nature of the relationship between attachment security and mindfulness (Shaver et al. 2007; Ryan et al. 2007), a bourgeoning body of literature has examined this relationship empirically. However, to the best of our knowledge, no systematic review of this literature has been conducted. It is therefore timely and useful to conduct a systematic review and statistical synthesis of the nature of the relationship between mindfulness and attachment. While much of the available literature tackles the question of “are they related?” more effectively than the question of “how are they related?,” it is our hope that the present review will serve as a springboard for future research to begin to tackle the important issue of mechanisms. In the current review, we therefore seek to (i) identify publications documenting the relationship between attachment style and mindfulness; (ii) synthesize the findings using meta-analysis; and (iii) critique the methodologies employed in order to make recommendations for future research.

## Method

### Search Strategy

A systematic search of the online databases PsycArticles, PsycInfo, PubMed, and Psychology and Behavioral Sciences Collection was conducted to find published articles. Unpublished works were searched using ProQuest. All searches were conducted between November 2015 and February 2016, using the combination of terms “attachment” and “mindfulness.” Forward and backward citation searching completed the search.

### Inclusion and Exclusion Criteria

Studies were included if they met the following criteria: (i) published/written in English. (ii) used a quantitative methodology, (iii) used psychometrically reliable and validated self-report measures of adult attachment and dispositional mindfulness, (iv) participants were aged 16 and over. For inclusion in the meta-analysis, studies were required to report the statistical association between attachment and dispositional mindfulness at one given time point (i.e., cross-sectional design, or baseline data). Single case designs were excluded. All authors contributed to the decision-making process for inclusion of articles; articles were only included if all authors were in agreement.

### Data Extraction

The following data were extracted from each study: country, year of publication, publication outlet, design, sample characteristics (gender, age, and, where available, meditation experience), design characteristics (sample population, measures used, experimental condition used, when applicable). Data pertaining to the statistical significance and effect size were also extracted from each study. The main aim of the meta-analysis was to assess the strength of the relationship between adult attachment dimensions and mindfulness (including subscales); therefore, we extracted statistics that detailed the nature of the relationship, which included *r* values (for use in the meta-analyses).

### Statistical Analysis

Twelve separate meta-analyses were performed, on data from 16 studies, to assess associations between adult attachment and dispositional mindfulness. The analyses evaluated the relationship between (1) attachment anxiety and total mindfulness and (2) attachment avoidance and total mindfulness. Further analyses (*n =* 10) were conducted on a subset of studies (*n* = ranged from 8 to 12) to assess the relationships between the two attachment dimensions (anxiety and avoidance) and five facets of mindfulness: acting with awareness, observing, describing, non-judging, and non-reacting subscales (Baer et al. 2006). All studies included in this analysis utilized the Five Facet Mindfulness Questionnaire (FFMQ; Baer et al. 2006), which includes each of the facets of mindfulness within five subscales, or the Kentucky Inventory of Mindfulness Skills (KIMS; Baer et al. 2004), which includes four of the five facets of mindfulness (excluding non-reactivity from those listed above). The remaining analyses therefore examined the relationships between (3) attachment anxiety and act with awareness; (4) attachment anxiety and observing; (5) attachment anxiety and describing; (6) attachment anxiety and non-judging; (7) attachment anxiety and non-reacting; (8) attachment avoidance and act with awareness; (9) attachment avoidance and observing; (10) attachment avoidance and describing; (11) attachment avoidance and non-judging; and (12) attachment avoidance and non-reacting.

The meta-analyses were conducted using STATA (version 12) and were based on random-effects models. Such models assume that the effect size of the relationship between the attachment and mindfulness variables in each of the studies varies as a function of differences in study characteristics as well as sampling error. The weighted average effect sizes were computed using the STATA command *metan* (Harris et al. 2008), which implements the random-effects model specified by DerSimonian and Laird (1986). Effect sizes were computed using Pearson’s *r* (*r*_+_). Standard errors, used to weight each effect size, were calculated according to the specifications of Lipsey and Wilson (2001). Effect sizes were interpreted using standard convention (Cohen 1992), in which values of .1, .3, and .5 represent small, medium, and large effect sizes, respectively.

### Moderator Analyses

Heterogeneity was evaluated using Cochran’s homogeneity *Q* statistic and *I* ^2^ statistic. In the event that the *Q* statistic is significant, this indicates that the relationship between the specified attachment and mindfulness variables across the relevant set of studies could be due to factors other than sampling error. The *I* ^2^ statistic is an estimate of the percentage of variability in the effect size estimate that can be attributed to these factors, as opposed to the sampling error. As a general guideline, an *I* ^2^ statistic of 30 to 60% indicates moderate variability, with over 75% indicating considerable variability (Higgins et al. 2009).

In order to assess whether certain characteristics of the included population samples moderated the relationship between adult attachment dimensions (anxiety and avoidance) and mindfulness, moderator analyses were conducted using a metaregression approach (Thompson and Sharp 1999). This method can be used to determine the effect of both continuous and categorical moderators in order to assess whether each moderator was associated with significant variance in the effect size for each reported relationship (the beta and *p* values in meta regression indicate the strength and significance of this association, respectively). These analyses were performed using the STATA command *metareg* (Hardboard and Higgins 2008). Moderators were coded across studies in order to characterize differences in study samples. These moderators focused on demographic characteristics of the included samples, more specifically the possible effects of mean age and the percentage of female participants.

### Quality Assessment

An assessment of the quality of included studies informed the critique of the literature and highlighted areas for future directions, rather than determining inclusion in the review. Papers were rated by the first author using an adapted form of the Effective Public Health Practice Project (EPHPP) Tool. The EPHPP has been shown to have good construct validity (Thomas et al. 2003) and inter-rater reliability (Armijo-Olivo et al. 2012). All studies were assessed on four relevant criteria taken from the EPHPP: (a) Selection bias, (b) Blinding, (c) Data collection methods, and (d) Withdrawals and dropout (attrition). Each domain is given an overall rating of “strong,” “moderate,” or “weak.” A global rating is then allocated on the following basis: strong (no weak ratings), moderate (one weak rating), or weak (two or more weak ratings).

## Results

**Fig. 1:**
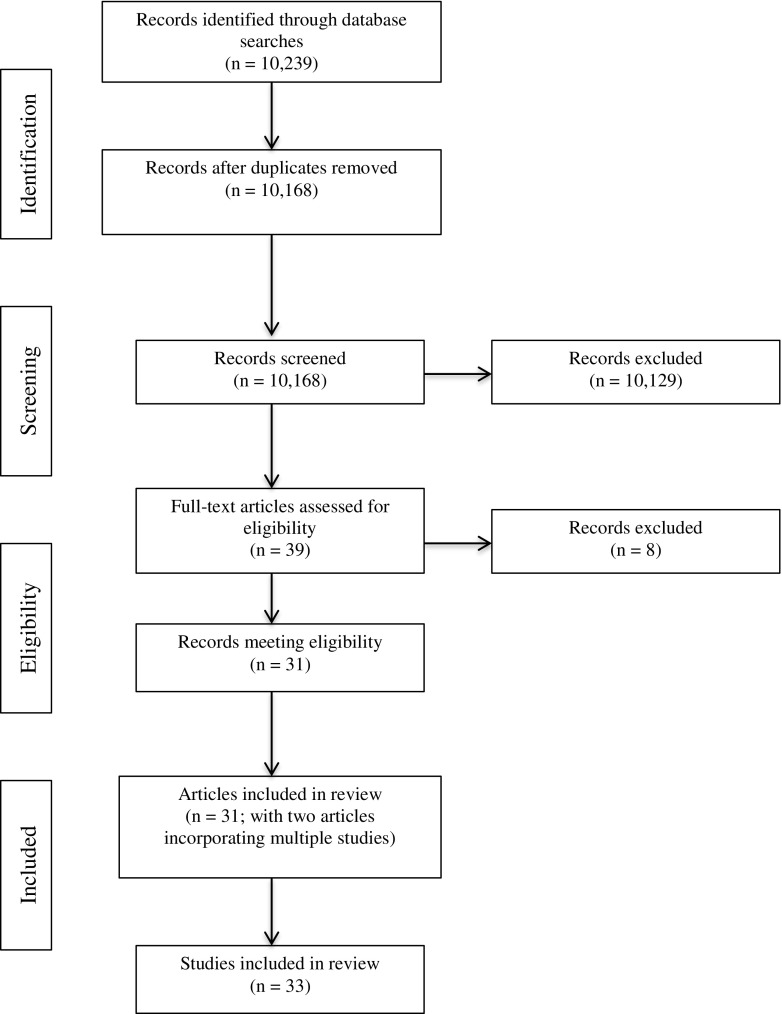
Flow diagram of systematic search

Initial searches yielded 10,239 papers published between 1919 and 2016. Seventy-one studies were duplicates. Therefore, 10,168 titles and abstracts were screened using the inclusion criteria. Thirty-nine full text articles were accessed, of which 31 fulfilled criteria for inclusion. Several articles reported multiple studies; only studies reporting on the relationship between adult attachment and mindfulness were extracted from these papers. A total of 33 studies were included in the review (see Fig. 1 for PRISMA diagram).

### Overview of Studies

Table 1 provides an overview of all studies. The studies were conducted across a number of countries: USA (*n* = 22), Australia (*n* = 6), UK (*n* = 3), Canada (*n* = 1), and Italy (*n* = 1). Sample size ranged from 39 to 1702, with a large portion of studies (*n* = 25) including over 100 participants. The majority of studies used undergraduate student (*n* = 20) samples, three of which used a mix of undergraduate students and the general population. One study used high school students over the age of 16.

**Table 1:** Studies exploring the association between adult attachment and mindfulness

Authors, year, and country	Participant sample	Procedure	Measure of attachment	Measure of mindfulness	Results	Quality rating
Caldwell and Shaver (2013); USA	93 undergraduate students	Completed measures at one time point	Experiences in Close Relationships Scale (ECR)	Mindful Attention Awareness Scale (MAAS)	Attachment anxiety and avoidance significantly negatively correlated with mindfulness	Moderate
Caldwell and Shaver (2015); USA	39 women who suffered childhood maltreatment	Mindfulness intervention, completed measures at multiple time points	Experiences in Close Relationship Scale (ECR)	Five Facet Mindfulness Questionnaire (FFMQ)	Attachment and avoidance significantly negatively correlated with total mindfulness	Strong
Ciano (2013); USA^a^	102 adults from the general population	Completed measures at one time point	Experiences in Close Relationships Scale–Revised (ECR-R)	Mindful Attention Awareness Scale (MAAS)	Attachment anxiety and avoidance significant predictor of the non-judge subscale	Strong
Cordon and Finney (2008); USA^a^	495 undergraduate students	Completed measures at one time point	Experiences in Close Relationships Scale–Revised (ECR-R)	Mindful Attention Awareness Scale (MAAS)	Attachment security associated with greater mindfulness	Moderate
Edwards (2014); USA	81 newlywed couples in 1st year of marriage	Completed measures at one time point	Experiences in Close Relationships Scale–Revised (ECR-R)	Mindful Attention Awareness Scale (MAAS)	For both husbands and wives, significant negative correlation between attachment anxiety and mindfulness	Moderate
Falb (2015); USA^a^	87 undergraduate students	Completed measures at three time points	Experiences in Close Relationships Scale–Revised (ECR-R)	Five Facet Mindfulness Questionnaire (FFMQ)	Secure attachment predicted mindfulness. Level of attachment predicted 4/5 mindfulness subscales (describing; act with awareness; non-judge; non-react)	Weak
Fossati et al. (2011); ITA^a^	501 high school students (16 years +)	Completed measures at one time point	Attachment Style Questionnaire (ASQ)	Mindful Attention Awareness Scale (MAAS)	Low mindfulness scores associated with aspects of avoidant and anxious attachment	Strong
Goodall et al. (2012); UK	199 adults from the general population	Completed measures at one time point	Experiences in Close Relationships Scale–Revised (ECR-R)	Five Facet Mindfulness Questionnaire (FFMQ)	Attachment anxiety significantly negatively correlated with subscales describe, act with awareness, non-judge, and non-react. Attachment avoidance significantly negatively correlated with subscales describe, act with awareness, and non-judge	Moderate
Hertz et al. (2015); USA^a^	103 undergraduate student couples	Completed measures at one time point	Experiences in Close Relationships Scale (ECR)	Five Facet Mindfulness Questionnaire (FFMQ)	Attachment anxiety and avoidance significantly negatively correlated with mindfulness. Mindfulness score significant in predicting attachment anxiety and avoidance	Strong
Authors, year, and country	Participant sample	Procedure	Measure of attachment	Measure of mindfulness	Results	Quality rating
Kubota (2014); USA	112 undergraduate students	Completed measures at one time point	Relationship Questionnaire (RQ) Experiences in Close Relationships Scale – Revised (ECR-R)	Five Facet Mindfulness Questionnaire (FFMQ) Mindful Attention Awareness Scale (MAAS)	Attachment anxiety significantly correlated with total mindfulness score and 4/5 subscales (describe; act with awareness; non-judge; non-react). Attachment avoidance significantly correlated with total mindfulness score and 3/5 subscales (describe; act with awareness; non-judge)	Moderate
Leigh (2010); USA	200 undergraduate students	Completed measures at one time point	Experiences in Close Relationships Scale (ECR)	Freiburg Mindfulness Inventory (FMI) Five Facet Mindfulness Questionnaire (FFMQ)	Attachment anxiety significantly negatively correlated with total mindfulness score and 3/5 subscales (act with awareness; non-judge; non-react). Attachment avoidance negatively correlated with total mindfulness score and 4/5 subscales (describe; act with awareness; non-judge; non-react). Attachment anxiety significantly predicted lower mindfulness and act with awareness, non-react, and non-judge subscales. Attachment avoidance significantly predicted lower mindfulness and describe subscale	Moderate
Ma (2008); USA^a^	90 undergraduate students seeking therapy within past 6 months	Completed measures pre and post (multiple time points) therapy	Experiences in Close Relationships Scale (ECR)	Five Facet Mindfulness Questionnaire (FFMQ)	Attachment security significantly correlated with mindfulness	Moderate
Macaulay et al. (2015); CAN	505 undergraduate students	Completed measures at one time point	Experiences in Close Relationships Scale–Revised (ECR-R)	Kentucky Inventory of Mindfulness Sills (KIMS)	Attachment anxiety significantly negatively correlated with 2/4 KIMS subscales (act with awareness; accept). Attachment avoidance significantly negatively correlated with describe subscale only	Weak
Authors, year, and country	Participant sample	Procedure	Measure of attachment	Measure of mindfulness	Results	Quality rating
Maniaci (2015); USA	175 heterosexual married couples	Completed measures at 4 time points (data extracted only from first)	Experiences in Close Relationships Scale–Revised (ECR-R)	Five Facet Mindfulness Questionnaire (FFMQ)	In husbands, attachment anxiety and avoidance significantly negatively correlated with mindfulness. In wives, only attachment anxiety significantly negatively correlated with mindfulness	Moderate
Martin (2012); USA^a^	Two samples—28 recruited from counseling services, 81 undergraduate students	Daily assessment battery of measures	Experiences in Close Relationships Scale (ECR) State Adult Attachment Measure (SAAM)	Five Facet Mindfulness Questionnaire (FFMQ) Cognitive and Affective Mindfulness Scale–Revised (CAMS-R)	Findings suggest increased mindfulness reduces attachment anxiety and help to reduce attachment avoidance tendencies	Moderate
Ormiston (2011); USA	300 individuals in relationship (at least 6 months in length)	Completed measures at one time point	Experiences in Close Relationships Scale–Revised (ECR-R) Relationship Questionnaire (RQ)	Mindful Attention Awareness Scale (MAAS)	Attachment anxiety and avoidance significantly negatively correlated with mindfulness	Moderate
Palmer (2014); USA	120 individuals from the local community	Completed measure pre and post positive recall intervention	Experiences in Close Relationships Scale (ECR)	Mindful Attention Awareness Scale (MAAS)	Attachment anxiety significantly positively correlated with mindfulness	Strong
Pepping et al. (2013); AUS	572 undergraduate students	Completed measures at one time point	Experiences in Close Relationships Scale–Revised (ECR-R)	Five Facet Mindfulness Questionnaire (FFMQ)	Attachment anxiety and avoidance significantly negatively correlated with mindfulness	Moderate
Pepping and Duvenage (2016); AUS^a^ Study 1	128 undergraduate students	Completed measures at one time point	Experiences in Close Relationships Scale–Revised (ECR-R)	Child and Adolescent Mindfulness Measure (CAMM)	Attachment anxiety and avoidance associated with lower mindfulness	Moderate
Pepping et al. (2013); AUS^a^	290 undergraduate students	Completed measures at one time point	Experiences in Close Relationships Scale–Revised (ECR-R)	Five Facet Mindfulness Questionnaire (FFMQ)	Attachment anxiety and avoidance significantly negatively correlated with total mindfulness. Significant differences between meditators and non-meditators. Across both samples (meditators and non-meditators) attachment anxiety and avoidance significantly associated with all 5 mindfulness subscales	Moderate
Authors, year, and country	Participant sample	Procedure	Measure of attachment	Measure of mindfulness	Results	Quality rating
Pepping et al. (2015); AUS Study 1 Study 2	144 undergraduate students 55 women seeking eating pathology treatment	Completed measures at one time point Completes measures at one time point	Experiences in Close Relationships Scale–Revised (ECR-R) Experiences in Close Relationships Scale–Revised (ECR-R)	Five Facet Mindfulness Questionnaire (FFMQ) Five Facet Mindfulness Questionnaire (FFMQ)	Attachment anxiety and avoidance significantly negatively correlated with mindfulness. Attachment anxiety and avoidance significantly negatively correlated with mindfulness	Moderate Moderate
Pidgeon and Giufre (2014); AUS^a^	156 undergraduate students	Completed measures at one time point	Experiences in Close Relationships Scale–Revised (ECR-R)	Freiburg Mindfulness Inventory (FMI)	Attachment anxiety and avoidance significantly negatively correlated with mindfulness	Moderate
Rowe et al. (2016); UK	117 participants; undergraduate students and non-students	Testing period of 4 weeks, self-compassion priming	Experiences in Close Relationships Scale (ECR)	Freiburg Mindfulness Inventory (FMI) Toronto Mindfulness Scale (TMS)	Attachment anxiety and avoidance not significantly correlated with trait or state mindfulness	Moderate
Saavedra (2011); USA Study 1 Study 2	1501 general population currently in romantic relationship 187 couples—two subsamples 89 couples from previous study and an additional 98 couples	Completed measures at multiple time points Completed measures at multiple time points	Experiences in Close Relationships Scale–Revised (ECR-R) Experiences in Close Relationships Scale–Revised (ECR-R)	Mindful Attention Awareness Scale (MAAS) Mindful Attention Awareness Scale (MAAS)	In both females and males, attachment anxiety and avoidance were significantly negatively correlated with mindfulness (labeled as act with awareness). In both female and males, attachment anxiety and avoidance were significantly negatively correlated with total mindfulness. Attachment anxiety significantly negatively correlated with subscales non-judge and non-react. Attachment avoidance significantly negatively correlated with subscales describe and non-judge	Moderate Moderate
Saavedra et al. (2010); USA	1702 individuals in a romantic relationship	Completed measures at one time point	Experiences in Close Relationships Scale–Revised (ECR-R)	Mindful Attention Awareness Scale (MAAS)	Attachment anxiety and avoidance significantly negatively correlated with mindfulness	Moderate
Sahdra et al. (2011); USA	60 individuals from general population recruited through meditation magazine	Completed measures before and after mindfulness intervention	Experiences in Close Relationship Scale (ECR)	Five Facet Mindfulness Questionnaire (FFMQ)	Attachment anxiety significantly negatively correlated with total mindfulness and subscales act with awareness, non-judge, and non-react. Attachment avoidance significantly negatively correlated with total mindfulness and all 5 subscales	Strong
Authors, year, and country	Participant sample	Procedure	Measure of attachment	Measure of mindfulness	Results	Quality rating
Somohano (2013); USA^a^	97 undergraduate students in relationship cohabiting with partner	Completed measures at one time point	Experiences in Close Relationships Scale–Revised (ECR-R)	Freiburg Mindfulness Inventory (FMI)	Significant difference in mindfulness scores between attachment groups. Clinical significance between secure and anxious attachment	Moderate
Tomac (2011); USA^a^	114 individuals from university participant pool	Completed measures at one time point	Experiences in Close Relationships Scale–Revised (ECR-R)	Mindful Attention Awareness Scale (MAAS)	Attachment security significantly negatively correlated with total mindfulness. Attachment anxiety and avoidance related to lower mindfulness scores	Moderate
Walsh et al. (2009); UK Study 1	127 undergraduates and university staff	Completed measures at one time point	Experiences in Close Relationships Scale–Revised (ECR-R)	Mindful Attention Awareness Scale (MAAS)	Attachment anxiety and avoidance significantly negatively correlated with mindfulness. 18% variance mindfulness scores accounted for when regressed onto attachment anxiety, avoidance, their interaction, and trait anxiety	Weak
Wang (2011); USA	282 undergraduate students	Completed measures at one time point	Experiences in Close Relationships Scale–Revised (ECR-R) The Inventory of Parent and Peer Attachment (IPPA)	Mindful Attention Awareness Scale (MAAS) Toronto Mindfulness Scale (TMS)	Attachment anxiety and avoidance significantly negatively correlated with mindfulness	Weak
Wilson (2012); USA	Undergraduate students 1st phase—315 2nd phase—232	Completed measures pre and post trauma writing intervention	Experiences in Close Relationships Scale (ECR)	Kentucky Inventory of Mindfulness Skills (KIMS)	Attachment anxiety significantly negatively correlated with total KIMS mindfulness, describe, act with awareness, and accept significantly positively correlated with observe subscale. Attachment avoidance significantly negatively correlated with total KIMS mindfulness, describe, act with awareness, and accept	Moderate

^a^Denotes studies *not* included in the meta-analyses

Five studies specifically recruited couples, including couples from the general population (*n* = 1), newlyweds within their first year of marriage (*n* = 1), heterosexual married couples (*n* = 1), undergraduate student couples (*n* = 1), and couples in a relationship lasting longer than 6 months (*n* = 1). Two studies recruited participants who were in or had recently been in a romantic relationship including individuals in a committed relationship greater than 1 year (*n* = 1), students who had experienced the dissolution of a romantic relationship in the last 24 months (*n* = 1). Three studies investigated populations that had sought out or were currently seeking psychological intervention (counseling and eating pathology treatment).

The majority of studies measured adult attachment using the Experiences in Close Relationships Revised scale (ECR-R; Fraley et al. 2000; *n* = 22). The ECR-R measure has been used extensively in attachment research and is a revised version of the original scale, which provides attachment scores along two dimensions (attachment anxiety and avoidance). Other measures included the Experiences in Close Relationships scale (Brennan et al. 1998; *n* = 10), the State Adult Attachment Measure (SAAM; Gillath et al. 2009; *n* = 1), the Attachment Style Questionnaire (ASQ; Feeney et al. 1994; *n* = 1), the Relationship Questionnaire (RQ; Bartholomew and Horowitz 1991; *n* = 1), and the Inventory of Parent and Peer Attachment (IPPA; Armsden and Greenberg 1989; *n* = 1).

The most commonly used measures of mindfulness were the Mindful Attention Awareness Scale (MAAS; Brown and Ryan 2003; *n* = 14) and the FFMQ (Baer et al. 2006; *n* = 14). Both measures provide a total score, representing overall trait mindfulness; the FFMQ also provides scores for five subscales (Observe, Describe, Act with awareness, Non-judge, Non-react). Other measures included the Freiburg Mindfulness Inventory (FMI; Walach et al. 2006; *n* = 4), the KIMS (Baer et al. 2004; *n* = 2), the Toronto Mindfulness Scale (TMS; Lau et al. 2006; *n* = 2), the Child and Adolescent Mindfulness Measure (CAMM; Greco et al. 2011; *n* = 1), and the Cognitive and Affective Mindfulness Scale-Revised (CAMS-R; Feldman et al. 2007; *n* = 1). It should be noted that several studies used multiple measures of adult attachment and mindfulness.

For the relationships between attachment dimensions and both total mindfulness and the subscales, we first present the meta-analysis findings followed by some observations from our narrative review.

### Relationship Between Attachment Dimensions and Total Mindfulness

Table 2 presents the results from each of the meta-analyses conducted. The relationships between the two attachment dimensions (anxiety and avoidance) and dispositional mindfulness both yielded small-to-medium effect sizes.

**Table 2:** Sample-weighted average effect size of the relationship between adult attachment and mindfulness variables

Relationship measured	*r*_*+*_	*k*	*n*	95% CI	*X* ^2^	*I* ^2^
Adult attachment anxiety						
Total mindfulness	−.360***	22	5964	−.40, −.32	60.92***	65.5%
Act with awareness	−.332***	12	5637	−.38, −.29	26.27**	58.1%
Observe	.013	10	2279	−.07, .10	37.95***	76.3%
Describe	−.169***	10	2279	−.26, −.08	42.86***	79%
Non-judge	−.451***	10	2279	−.51, −.40	19.20**	53.1%
Non-react	−.258***	8	1542	−.35, −.16	25.90**	73%
Adult attachment avoidance						
Total Mindfulness	−.281***	21	5844	−.33, −.23	73.21***	72.7%
Act with awareness	−.258***	12	5637	−.31, −.20	42.60***	74.2%
Observe	−.091*	10	2279	−.17, −.02	28.03**	67.9%
Describe	−.285***	10	2279	−.37, −.20	36.65***	75.4%
Non-judge	−.275***	10	2279	−.32, −.21	22.92**	60.7%
Non-react	−.162**	8	1542	−.27, −.05	33.83***	79.3%

*CI* confidence interval

*p < .05; **p < .01; ***p < .001

The overall sample-weighted relationship between attachment anxiety and mindfulness was *r*_+_ = −.36 (95% CI [−.40, −.32]), based on 22 participant samples taken from 19 articles and 5964 participants. There was significant variation in the observed relationship across studies (*Q*[22] = 60.92, *p* < .001), with a moderate-to-high level of heterogeneity across studies (*I* ^2^ = 65.5%). The majority of coefficients reported in the studies were significant and negative, ranging −.22 to −.63 (Palmer 2014; Pepping et al. 2013, respectively).

The overall sample-weighted relationship between attachment avoidance and mindfulness was *r*_+_ = −.28 (95% CI [−.33, −.23]), based on 21 participant samples taken from 18 articles, and 5844 participants. There was significant variation in the observed relationship across studies (*Q*[21] = 73.21, *p* < .001), with a high level of heterogeneity across studies (*I* ^2^ = 72.7%). The majority of coefficients reported in studies were significant and negative, ranging from −.21 to −.54 (Pepping et al. 2013; Wilson 2012).

Contradictory to the above findings, Rowe et al. (2016) was the only study that did not find any significant correlations between attachment and mindfulness (attachment anxiety-mindfulness, *r* = .12, −.10; attachment avoidance-mindfulness, *r* = −.14, −.20).

### Subscale Analysis

Subscale analysis examine the relationship between each of the two attachment dimensions (anxiety and avoidance) and the subscales of mindfulness (as measured by FFMQ and KIMS) produced nine (from a possible ten) significant, negative effect sizes (*p* < .05), of which three were small (anxiety-describe; avoidance-observe; avoidance-nonreact); five were medium (anxiety-act with awareness; anxiety-nonreact; avoidance-act with awareness; avoidance-describe; avoidance-nonjudge); and one was large (anxiety-nonjudge). Thus, higher levels of attachment insecurity (avoidance or anxiety) were associated with lower levels of dispositional mindfulness on almost every dimension (bar attachment anxiety and observe, see Table 2).

The largest effect size was between attachment anxiety and “non-judge”, reflecting that the strongest significant correlations were consistently reported between attachment anxiety and non-judging (range *r* = −.33 to −.61; both reported in Pepping et al. 2014). The relationships between attachment dimensions and the “observe” subscale were the weakest, with the effect size for avoidance-“observe” being the smallest significant effect, and the effect size for anxiety-“observe” being non-significant. Across the studies, the observe subscale of mindfulness was widely reported as being negatively and non-significantly associated with both dimensions of adult attachment in all but three studies (Pepping et al. 2014; Sahdra et al. 2011; Wilson 2012). These studies reported significant positive associations between the observe subscale and attachment anxiety (range *r* = .14 to .15) and significant negative associations between the observe subscale and attachment avoidance (range *r* = −.27 to −.30).

### Moderators of the Relationship Between Adult Attachment and Mindfulness

Two population sample characteristics were evaluated as moderators of the relationship between adult attachment dimensions (anxiety and avoidance) and total mindfulness (see Table 3).

**Table 3:** Moderators of the relationships between adult attachment dimensions and mindfulness

Relationship	Moderator	Regression coefficient	Standard error	*k*	*n*	95% CI	*I* ^2^	Adj *R* ^2^
Attachment anxiety and total mindfulness	Mean age	−.0003	.003	21	5837	−.007, .006	68.61%	−6.74%
	Percentage female	−.0009	.002	18	4940	−.004, .003	68.92%	−4.20%
Attachment avoidance and total mindfulness	Mean age	−.001	.004	20	5717	−.009, .007	75.33%	−7.22%
	Percentage female	−.001	.001	17	4820	−.004, .002	56.02%	1.19%

Columns *k* and *n* represent number of studies and number of participants, respectively. Additionally, studies that failed to report mean age or that reported male and female participant data separately were excluded from these analyses

*p < .05; **p < .01; ***p < .001

The metaregression confirmed that the mean age of participants did not moderate the observed effect size of the relationship between attachment anxiety and total mindfulness (*β* = −.0003, *p* = .906) or attachment avoidance and total mindfulness (*β* = −.001, *p* = .755). Likewise, the gender of participants did not moderate the observed effect sizes (attachment anxiety and mindfulness, *β* = −.0009, *p* = .576; attachment avoidance and mindfulness, *β* = −.001, *p* = .395). It can therefore be concluded that age and gender had no impact on effect sizes and that the variance in the measured relationships occurs irrespective of these sample characteristics.

### Methodological Critique

Overall quality ratings from the EPHPP assessment are provided in Table 1; domain specific ratings are reported in Table 4. A large majority (23 out of 33) of the reviewed studies were rated as moderate, while only six were rated as strong. A major weakness across studies was selection bias. Almost all of the studies failed to include a representative sample, with the majority sampling a student population (*n* = 22), which limits the generalizability of the findings to a wider population.

**Table 4:** Quality ratings (weak, moderate, and strong) for the adapted EPHPP and overall quality rating

Authors (date)	Selection bias	Blinding	Measures	Attrition	Overall
Caldwell and Shaver (2013)	M	M	S	N/A	M
Caldwell and Shaver (2015)	S	M	S	S	S
Ciano (2013)	S	W	S	N/A	S
Cordon and Finney (2008)	W	M	S	N/A	M
Edwards (2014)	M	W	S	N/A	M
Falb (2015)	W	W	S	W	W
Fossati et al. (2011)	S	W	S	N/A	S
Goodall et al. (2012)	M	W	S	N/A	M
Hertz et al. (2015)	S	W	S	S	S
Kubota (2014)	M	W	S	N/A	M
Leigh (2010)	M	W	S	N/A	M
Ma (2008)	M	W	S	N/A	M
Macaulay et al. (2015)	W	W	S	N/A	W
Maniaci (2015)	M	M	M	W	M
Martin (2012)	M	W	S	M	M
Ormiston (2011)	M	W	S	N/A	M
Palmer (2014)	M	S	S	S	S
Pepping and Duvenage (2016)					
Study 1	W	M	S	N/A	M
Pepping et al. (2013)	W	M	S	N/A	M
Pepping et al. (2013)	W	M	S	N/A	M
Pepping et al. (2015)					
Study 1	W	M	S	N/A	M
Study 2	M	M	S	N/A	M
Pidgeon and Giufre (2014)	W	M	S	N/A	M
Rowe et al. (2016)	M	M	S	W	M
Saavedra (2011)					
Study 1	M	M	S	W	M
Study 2	M	M	S	W	M
Saavedra et al. (2010)	M	M	S	N/A	M
Sahdra et al. (2011)	S	M	S	S	S
Somohano (2013)	M	W	S	N/A	M
Tomac (2011)	M	M	S	N/A	M
Walsh et al. (2009)	W	W	S	N/A	W
Wang (2011)	W	S	W	N/A	W
Wilson (2012)	W	M	S	N/A	M
Total for each dimensions					
Weak	11	14	1	5	4
Moderate	17	17	1	1	23
Strong	5	2	31	4	6

Strong = 3+ strong ratings. Moderate = 2+ moderate/strong, <2 weak. Weak = 2+ weak ratings

There were also inherent limitations in the design of the studies reviewed. By virtue of the aims of this review, and the inclusion criteria, all of the studies relied on a cross-sectional design, which saw participants completing self-report measures of attachment and mindfulness at one time point. While an appropriate way in which to capture data on the relationship between two variables, this study design does not allow for inferences about causality or confirm stability of any identified relationship over time. Furthermore, while the measures included were reliable and validated, it should not be ignored that the self-report nature of these measures could lead to potential response bias. The majority of studies used the ECR-R to measure adult attachment, which is multi-dimensional, measuring individual differences in attachment anxiety and attachment avoidance, whereas some studies used a state measure of adult attachment (studies examining state mindfulness were not included in the meta-analyses). It can be argued that the ECR-R, while a validated and reliable measure, is considered to measure adult attachment as a trait, similar to a personality trait. Studies using different measures of attachment and mindfulness reported similar associations, despite the lack of consistency in measurement.

## Discussion

The current review presents the first systematic synthesis, meta-analysis, and critical appraisal of existing research that has set out to examine the association between adult attachment dimensions and mindfulness. We here discuss the key findings in relation to theory, methodological issues relating to the literature reviewed, limitations, and implications for future research.

Using meta-analysis, we found a clear significant relationship between the two constructs, with anxiety and avoidance attachment dimensions being associated with, and in some cases, statistically predictive of, levels of total mindfulness. A large majority of the cross-sectional studies included in the review reported significant negative correlations between attachment anxiety and mindfulness. However, Edwards (2014), Maniaci (2015), and Rowe et al. (2016) failed to find significant associations between attachment avoidance and total mindfulness scores. Interestingly, both Edwards (2014) and Maniaci (2015) reported non-significant correlations for the females taking part in the study, while only Edwards (2014) reported non-significant correlations for both male and female participant mindfulness scores and attachment avoidance. Rowe et al. (2016) did not report significant associations between attachment anxiety and state and trait mindfulness. However, this appears to be an anomalous result when compared with the majority of the studies included in the review. The remaining cross-sectional studies reported significant correlations between adult attachment avoidance and mindfulness scores for both males and females (where applicable).

Interestingly, attachment anxiety was more often negatively associated with total mindfulness than attachment avoidance; this was also reflected by the results of the meta-analyses. In line with attachment theory (Mikulincer and Shaver 2007), it appears as though individuals higher in attachment anxiety exhibit a hyperactivation of the attachment system which may, in turn, hinder the optimal fostering of underpinning constructs of mindfulness (such as acting with awareness, non-judging, and also non-reacting). Hypervigilance to threat might also explain the occasional positive association between attachment anxiety and the observe subscale of mindfulness (Ryan et al. 2007). Hypervigilant individuals may be inclined to notice threat cues more readily, which predisposes them to observe and attend to the situations they experience significantly more so than individuals with higher levels of attachment avoidance (Ryan et al. 2007). Meanwhile, those individuals high in attachment avoidance exhibit discomfort with closeness and dependency and so tend to minimize this discomfort by way of deactivation of the attachment system and decreased observation to threats. However, there are important caveats regarding the observe subscale, which mean that caution is needed to not over-interpret any findings pertaining to it (especially in relation to attachment anxiety, where the meta-analysis found no significant relationship with it).

Baer et al. (2006) reported that while observing as a central tendency of mindfulness, the subscale failed to fit their proposed Confirmatory Factor Analysis model, which could be attributed to the differential correlations between observe and the other four facets. They proposed that the emphasis the observe subscale places on external stimuli does not adequately capture the quality of noticing/attending to experience (Baer et al. 2006). Additionally, they reported a significant negative correlation between observe and non-judge subscales. It would appear that meditation practice and experience has an impact on the individual facets of mindfulness among individuals. It was suggested that in individuals with no meditation experience and attending to experiences may be associated with judging them but, through meditation experience, should be expected to exhibit higher levels of observing and non-judging. Pepping et al. (2014) reported differences among the mindfulness subscales between meditators and non-meditators. More specifically, among non-meditators, attachment anxiety and avoidance were positively correlated with observing, whereas for meditators this relationship was negative. There appears to be contention regarding the efficacy of the observe subscale of mindfulness, as it may tap different things depending on the sample, for example when comparing experienced vs. non-experienced meditators and clinical vs. non-clinical participants (Grossman and Van Dam 2011). Future research should aim to examine the relationship between attachment and the observe facet of mindfulness with an improved measurement tool.

Overall, attachment anxiety was significantly negatively correlated with total mindfulness, as well as four of the five subscales of the mindfulness subscales (act with awareness, describe, non-judge, and non-react). The strongest association was between attachment anxiety and the non-judge subscale across the three most commonly used mindfulness measures; the “non-judge” subscale of the FFMQ, the “accept without judgment” subscale of the KIMS, and the “non-judging” subscale of the MAAS (reported in Macaulay et al. 2015; Pepping et al. 2014; Saavedra 2011). Additionally, the meta-analyses and synthesis highlight the significant negative relationships between attachment avoidance, total mindfulness, and each of the five mindfulness subscales. The “describe” and “non-react” subscale of the FFMQ and the “describe” subscale of the KIMS reported the strongest correlations with attachment avoidance (reported in Pepping et al. 2014; Macaulay et al. 2015). The deactivation of the attachment system exhibited by individuals high in attachment avoidance (Mikulincer and Shaver 2007) can explain these relationships. Those high in avoidance tend to cut off from their emotions (Wei et al. 2005). Although this might suggest that avoidant individuals would therefore be better able to be non-reacting, ironically, their emotional cutoff then leads them to experience greater negative mood (Wei et al. 2005). Furthermore, while avoidant individuals are typically good at suppressing unwanted thoughts (Mikulincer et al. 2004), these strategies are known to fail under cognitive load (Mikulincer et al. 2004, Mikulincer et al. 2000), potentially undermining any facilitating effects on non-reacting they may have had.

While a majority of these studies reported the associations between attachment dimensions and total mindfulness, they still tell us little about the mechanisms of this association. However, Pepping et al. (2013) attempted to further understand this and reported that difficulties in emotion regulation fully mediated the relationship between adult attachment variables and mindfulness. However, this analysis was not conducted in the opposite direction, meaning that we cannot infer that attachment style leads to emotion regulation, which leads to mindfulness, rather than the other way around. Further research is needed to address directionality.

As reported above, the key limitations of the available literature linking adult attachment and mindfulness is firstly the reliance on cross-sectional data collection, and secondly the paucity of representative samples. While cross-sectional studies are invaluable for taking a first look at the nature of a relationship between two constructs (as was the focus of the present review), future research needs to move beyond this towards longitudinal data collection over time, in order to address the issues of development and causality. Based on the current state of the literature, it remains unclear whether increased levels of adult attachment anxiety and avoidance lead to increased mindfulness or vice versa. Several studies used regression analyses to further investigate the reported relationship, for example, Caldwell and Shaver (2013), found that attachment anxiety and avoidance were successful in significantly predicting higher levels of total mindfulness, although not over time, and others have begun to explore mechanisms (Macaulay et al. 2015). Nevertheless, further research is needed to expand on these findings and to fully explore the nature in which adult attachment and mindfulness are related and how they influence one another.

There is no universal measure of either adult attachment or mindfulness, which has led to a degree of measurement heterogeneity across studies. This means that there is no reason to believe that the findings presented in the included studies were a result of specific measures used. Equally, because similar findings emerged from studies of varying quality (ranging from weak to strong on the EPHPP), it is likely that the findings are not attributable to study quality. These two things combined give confidence in the overall findings as they appear to be both measure- and study quality-independent.

Furthermore, all studies included in the meta-analysis employed self-report measures of the constructs. Although the majority of the measures used are reliable and validated, a problem of the reliance on self-report methodology is that correlations between measures may be artificially inflated by shared method variance. Future research would benefit from including measures based on diagnostic interviews to further control for possible self-report biases.

### Limitations and Future Directions

While offering the first meta-analysis of the relationship between adult attachment style and mindfulness, the current review is not without limitations. Only articles published in the English language were included and while measures of adult attachment and mindfulness have been translated into other languages, it was not practical to include the research that employs them in the present review. This may have lead to an under-representation of certain cultures, potentially leading to generalizability issues.

The results of the metaregression indicated that neither mean age nor gender of participants were significant moderators of the effect size of the relationship between both adult attachment dimensions and total mindfulness scores. Therefore, these sample characteristics cannot account for the reported variance in the examined relationships. While the present study focused on these two key characteristics of the included sample populations and found there to be no significant effects, additional moderator analyses would be desirable. However, the studies reviewed proved too heterogeneous for further moderator analyses to be an option at present. That is to say, the variability of sample characteristics is too great to establish additional key variables to treat as potential moderators, and, crucially, to have a sufficient number of studies featuring each key variable. In future, as the literature grows it would be sensible to examine variables such as meditation experience, design characteristics, nationality of population sample, as well as the specific self-report measures used as potential moderators of the relationship between attachment style and mindfulness.

It is still largely unknown how mindfulness develops, whether it is a direct result of specific attachment styles or whether the core qualities of mindfulness influence the development of a secure attachment and overall adaptive functioning and to what extent. The current review is also limited by the quality of the studies reviewed, and their reliance on cross-sectional designs. Future research should seek to examine the development of attachment styles and mindfulness over time, as well as the extent to which one construct predicts another, and the mechanisms of these effects. Longitudinal design employing measurement of potential mediators would be a fruitful addition to current research. In conducting our review, we particularly noticed the paucity of research examining experimental manipulations or interventions targeting one construct, and measuring the outcomes on the other construct. In addition to longitudinal design, such work would go a long way to addressing causality and mechanisms, without the sometime prohibitive overheads of long-term longitudinal designs.

Additionally, the majority of studies examined here focused on the dispositional nature of both adult attachment and mindfulness. To further understand the relationship between and development of both constructs research may benefit from a shift to focusing on the state/contextual nature of both adult attachment and mindfulness. More specifically, to avoid further self-report biases, research may wish to employ more observational research methods. While there are observational methods available to measure adult attachment (e.g., the Adult Attachment Interview; George et al. 1985), no observational method exists to successfully assess mindfulness. Future research may wish to explore the development of an observational mindfulness assessment although this may prove difficult due to the inherently intrinsic qualities of the construct.

It is hoped that the present review and meta-analysis will serve as a spring broad for further research to address issues of causality and interaction between attachment and mindfulness. When the literature matures to include more prospective and experimental designs, a further review would be timely. It might be that targeting both variables could lead to even greater benefit than targeting one or the other, in which case the development and implementation of mindfulness and attachment-based interventions to enhance positive functioning and well-being could be improved.
